# Trihalomethane Cancer Risk Assessment for Private and Shared Residences: Addressing the Differences in Inhalation Exposure

**DOI:** 10.3390/toxics11040295

**Published:** 2023-03-23

**Authors:** Naseeba Parveen, Sudha Goel

**Affiliations:** 1 School of Environmental Science and Engineering, Indian Institute of Technology Kharagpur, Kharagpur 721302, West Bengal, India; t.parveennaseeba@gmail.com; 2 Department of Civil Engineering, Indian Institute of Technology Kharagpur, Kharagpur 721302, West Bengal, India

**Keywords:** inhalation risk, dermal contact, health analysis, water quality, carcinogens, probabilistic model, Monte Carlo simulation, risk assessment

## Abstract

The multi-pathway cancer risk (CR) assessment of trihalomethanes (THM) involves considering exposure via ingestion, dermal contact, and inhalation. Inhalation occurs during showering due to the volatilization of THMs from chlorinated water to the air. When assessing inhalation risks, exposure models commonly assume that the initial THM concentration in the shower room is zero. However, this assumption is only valid in private shower rooms where single or infrequent showering events take place. It fails to account for continuous or successive showering events in shared showering facilities. To address this issue, we incorporated the accumulation of THM in the shower room air. We studied a community (population ≈ 20,000) comprising two types of residences with the same water supply: population A with private shower rooms, and population B with communal shower stalls. The total THM concentration in the water was 30.22 ± 14.45 µg L^−1^. For population A, the total CR was 58.5 × 10^−6^, including an inhalation risk of 1.11 × 10^−6^. However, for population B, the accumulation of THM in the shower stall air resulted in increased inhalation risk. By the tenth showering event, the inhalation risk was 2.2 × 10^−6^, and the equivalent total CR was 59.64 × 10^−6^. We found that the CR significantly increased with increasing shower duration. Nevertheless, introducing a ventilation rate of 5 L s^−1^ in the shower stall reduced the inhalation CR from 1.2 × 10^−6^ to 7.9 × 10^−7^.

## 1. Introduction

Trihalomethanes (THM) are the most common chlorinated disinfection by-products. They are generally found in chlorinated tap water and are known to pose cancerous as well as non-cancerous health hazards [1,2,3]. Trichloromethane (TCM), bromodichloromethane (BDCM), dibromochloromethane (DBCM), and tribromomethane (TBM) are the four THM that are regulated in drinking water. As per the U.S. Environmental Protection Agency Integrated Risk Information System (USEPA-IRIS), TCM, BDCM, and TBM are probable (Group B2) and DBCM is a possible (Group C) carcinogen with either hepatic, urinary, or gastrointestinal systems as their target tumor sites [4]. Additionally, THM can cause non-cancerous health problems such as fatty cyst formation in the liver due to TCM, hazards to the urinary system due to BDCM, and adverse effects on the hepatic system due to both DBCM and TBM [4].

Several studies have investigated the concentration of THM in tap water around the world. In the United States, the maximum contaminant level for THM in drinking water is 80 µg L^−1^ as per the Safe Drinking Water Act enforced by the USEPA [5]. Similarly, Canada’s drinking water guidelines recommend a maximum concentration of 100 µg L^−1^ for THM [6]. The Indian Standards recommend individual guideline values of 200, 60, 100, and 100 µg L^−1^ for TCM, BDCM, DBCM, and TBM, respectively [7]. However, in many countries, THM concentrations in tap water can exceed these limits due to inadequate water treatment facilities and a lack of regulation. For instance, a study in Hong Kong found that the average concentration of THMs in tap water (n = 57) was 15.8–87.2 µg L^−1^ [8]. Wang et al., 2019, conducted an extensive survey on THM concentrations in drinking water in Jiangsu Province, China, during 2013–2015 (n = 485) and reported a range of 18.81 to 38.96 µg L^−1^ for the average THM concentration [9]. Similarly, (Furst et al., 2019, [10]) reported an average THM concentration of 68–99 µg L^−1^ in tap waters of three locations of Rajasthan, India [10]. The average concentration of THMs in Toronto, Canada, was 11.9 µg L^−1^, with a range of 3.7–20.0 µg L^−1^, and that in Dhahran, Saudi Arabia, was in the range of 1.7–9.4 µg L^−1^ with an average of 6.2 µg L^−1^ [11]. These studies demonstrate that the THM concentration in household tap water significantly varies between different regions worldwide.

THM cancer risk (CR) is individually calculated for three different exposure pathways: CR through ingestion, dermal absorption, and inhalation [12]. The sum of these pathways is used to determine the total CR [13]. Typically, risk assessment studies have assumed the initial pollutant concentration in shower air for inhalation exposure as ‘absent’ and it has been assumed to be zero in the calculations [11,14,15]. However, THMs are volatile compounds, and their concentration inside a shower stall can increase after each showering event, especially with frequent and continuous usage. This phenomenon is particularly relevant in common shower facilities, such as those found in student hostels, dormitories, public swimming pools, gymnasiums, small and medium lodges with communal bathrooms, and sports complexes, where there is often little time between successive showers to diminish the THM concentration left by the previous user. As a result, the concentration of THMs in the air needs to be accounted for when assessing the CR from exposure to total THM. A recent study evaluated the inhalation risk associated with continuous showering events and found that it increased with successive events [16]. However, this study did not assess the effect of increasing inhalation risk on the total CR.

The objective of the current study was to incorporate the THM concentration in the common shower rooms due to successive showers into a probabilistic risk assessment model. The study specifically aimed to determine the CR from exposure to THM for a community with two different types of residences: individual houses equipped with private shower rooms and hostels where the shower rooms are communal. Furthermore, the study investigated the effect of shower duration and ventilation rate on inhalation exposure.

## 2. Materials and Methods

### 2.1. Study Area and Population

Water samples were collected from 26 locations throughout the Indian Institute of Technology Kharagpur (IITKgp) campus. IITKgp is a residential educational institution with a population of approximately 20,000 individuals. A conventional water treatment plant (WTP) supplies treated water to the campus, which is chlorinated to maintain a free chlorine residual of 0.5 mg L^−1^ at the WTP outlet and 0.2 mg L^−1^ at the furthest consumer point. The campus is comprised of two distinct populations: (A) long-term residents who are faculty and staff, and (B) temporary residents who are students. The dwellings of these two populations differ; population A resides in family quarters or apartments with private bathrooms, while population B is accommodated in hostels with communal bathrooms/showers. To account for these differences, sampling locations were chosen from both family quarters and hostels.

### 2.2. Water Quality Analysis

Tap water from the washroom was collected in triplicates from all locations in brown glass vials. Before sampling, sodium sulfate (Merck Lifesciences, Bengaluru, India, 95.5%) was added to the sampling vials to quench any free residual chlorine. The samples were then transferred to the laboratory and stored at 4 °C until analysis. The pH of the water samples was measured using a table-top pH meter (Labtronics, Haryana, India). Free residual chlorine was determined using a portable diethyl-paraphenylene-diamine (DPD) colorimetric kit (DR300, Hach, Loveland, CO, USA). Anion concentrations were measured using ion chromatography (IC) (ICS2100, Thermo Fisher Scientific, Waltham, MA, USA). The total organic carbon (TOC) in the samples was analyzed using a TOC analyzer based on the heated per-sulfate wet oxidation method (Aurora 1030, OI Analytical, TX, USA).

### 2.3. Trihalomethane Analysis

A gas chromatograph (GC; Trace 1300, Thermo Fisher Scientific, Waltham, MA, USA) equipped with an electron capture detector (Ni^63^, ECD) and a silica fused capillary column (TG-5MS; 0.25 mm × 0.25 µm × 30 m) was used for the detection of THMs. Nitrogen (99.9% purity, Echo Gas, Kolkata, India) at a rate of 1.2 mL/min was used as carrier gas. The sample injection volume was 2 µL. The inlet and detector temperatures were 265 °C and 300 °C, respectively. The USEPA Method 501.1 with few modifications was followed for measuring THM concentration [17]. The oven temperature program of the GC system was 35 °C for 1 min, 35 to 40 °C at 1 °C/min, 40 °C to 200 °C at 80 °C/min, and 200 °C for 2 min. For the preconcentration of samples, a purge and trap unit (P&T) (Lumin, Teledyne Tekmar, Mason, OH, USA) was used. The P&T unit program was an 11-min purge followed by a 1-min dry purge, a 2-min desorb, and a 2-min bake.

Analytical standards of TCM, BDCM, DBCM, and TBM (99.9%, Sigma Aldrich, Bengaluru, India) were mixed in methanol (Merck Life Science, Bengaluru, India) to make 1000 mg L^−1^ of stock THM standard. Aqueous working standards of mixed THMs were made daily from the stock standard. Calibration standards were in the concentration range of 1 µg L^−1^ to 100 µg L^−1^. The calibration and final THM concentrations in samples were calculated using the Chromeleon™ Chromatography Data System (Chromeleon™ 7.2) software. Quality check standards of 2 µg L^−1^ and 10 µg L^−1^ were made daily from 1 mg L^−1^ mixed standard in THM free water and were periodically run. The retention time of TCM, BDCM, DBCM, and TBM was 4.44 min, 6.50 min, 8.76 min, and 10.76 min. The minimum detection limit of TCM, BDCM, DBCM, and TBM was 0.1, 0.25, 0.7, and 0.1 µg L^−1^. The limit of quantification was 1 µg L^−1^ for TCM and TBM, 1.5 µg L^−1^ for BDCM, and 2 µg L^−1^ for DBCM. Recoveries for 10 replicates of 2 µg L^−1^ for the four THMs were 100.2 ± 0.53%, 99.1 ± 0.80%, 103.3 ± 0.75%, and 98.8 ± 0.25% for TCM, BDCM, DBCM, and TBM, respectively.

### 2.4. Exposure Routes and Risk Assessment Models

**Figure 1:**
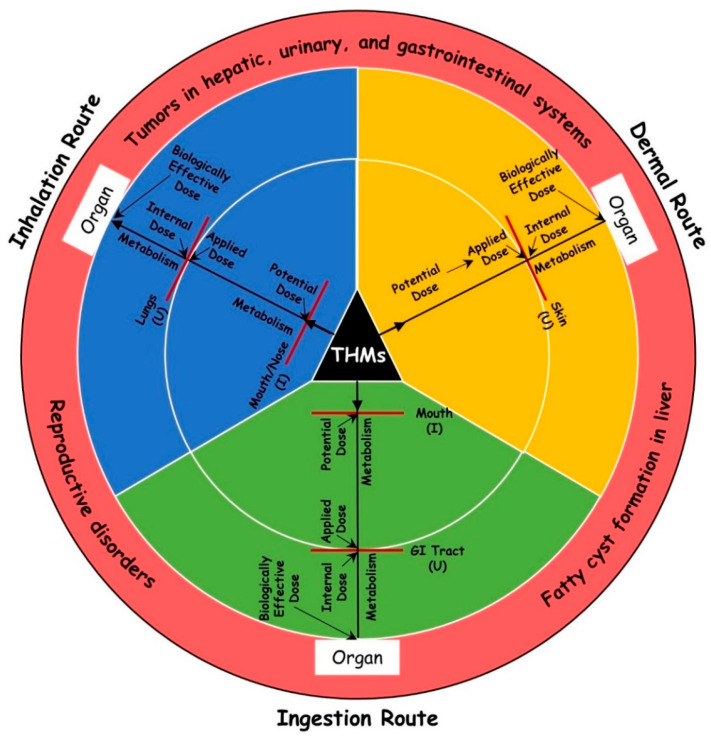
Schematic of source-to-effect of three exposure pathways of THM in human exposure models. Red lines are either exposure surface (a surface on a receptor where an agent is present) or absorption barrier (any exposure surface that can retard the rate of penetration of an agent into a receptor). I: intake; U: uptake; potential dose: the quantity of agent that go into a receptor after passing an exposure surface that is not an absorption barrier; applied dose: the amount of agent at an absorption barrier; internal dose: the amount of agent that enters a receptor by crossing an exposure surface acting as an absorption barrier; biologically effective dose: the quantity of agent that reaches the target internal organ or tissue where the harmful outcomes arise.

USEPA guidelines for CR assessment were adopted with several modifications. Three exposure routes were based on the source-to-outcome of THM (Figure 1). The present study focuses on CR assessment through these pathways, which was determined after calculating the daily intake dose of THM, as shown below:

(1) CR=CDI×CSF

where *CR* is the cancer risk, *CDI* is the chronic daily intake of THM (mg kg^−1^ day^−1^), and *CSF* is the cancer slope factor associated with each THM ((mg kg^−1^ day^−1^)^−1^).

The most used approach to calculate CDI using USEPA empirical formulae is a deterministic one, while a probabilistic approach has been infrequently used in the literature [18]. The probabilistic approach involves assigning statistical distributions for input variables to account for the associated uncertainty and variability [9,19]. The USEPA-recommended Monte Carlo simulation for risk assessment has been preferred by many researchers [20,21,22,23]. Ingestion rate, body weight, water temperature, and exposure duration are among the highly variable input parameters in the USEPA method, and accounting for their variability is crucial to evaluate their impact on the output parameter. A probabilistic risk assessment outputs a statistical distribution of intake doses or CR for a population. In this study, triangular distributions were employed for input variables, using minimum, most likely, and maximum values obtained from previous studies on the Indian population. The risk calculation input parameter details, the distribution and range of values, and their references are tabulated in Table 1. To address uncertainty in risk assessment, 10,000 iterations of Monte Carlo simulations were generated using the Companion software by Minitab™ [24].

**Table 1:** Model input parameters and their distribution.

Parameters	Distribution and Values	Reference
*Cwi,*/*Chw*—THM concentration (µg L^−1^/µg m^3^)	Table 2	This study
*IR*—Ingestion rate (L day^−1^)	T ^a^ (1, 2, 3)	[25]
*Pd*—Permeability of THM through the skin (m min^−1^)	T (0.0000267, 0.00003, 0.000035)	[26]
*t*—Showering duration (minutes)	T (5, 10, 15)	[11]
*Vs*—Shower stall volume (L)	T (2000, 3500, 5000)	[27]
*H*—Henry’s constant	TCM: 0.25, BDCM: 0.124, DBCM: 0.0526, TBM: 0.0501.	[14]
*Qw*—Water flow (L min^−1^)	T (3, 4, 5)	[16]
*Qg*—Air flow/ventilation rate (L min^−1^)	T (40, 50, 60)	[1]
*KoLA*—Overall mass coefficient of each THM (L min^−1^)	TCM: 7.4, BDCM: 5.9, DBCM: 4.6, TBM: 3.7	[28]
*R*—Breathing rate (m^3^ min^−1^)	T (0.012, 0.014, 0.016)	[14]
*F*—Showering frequency (events day^−1^)	T (0.72, 0.74, 0.76)	[11]
*EF*—Exposure frequency (days year^−1^)	T (330, 350, 365) drinking, T (300, 330, 365) dermal, T (300, 330, 360) inhalation	[1]
*ED*—Exposure duration (year)	Female: T (67, 72, 77) Male: T (65, 70, 75)	[29]
*BW*—Bodyweight (kg)	Female: T (50, 55, 60) Male: T (60, 65, 70)	[30]
*AT*—Averaging time (day)	Female: T (24,455, 26,280, 28,105) Male: T (23,725, 25,550, 27,375)	Calculated as per exposure duration
*T*—water temperature (°C)	Cold: T (10, 15, 20) Hot: T (35, 40, 45)	[16]
*CSF*—Cancer slope factor (mg kg^−1^ day^−1^)		[1]
Trichloromethane	Oral and dermal: 0.0061	
Bromodichloromethane	Oral and dermal: 0.062	
Dibromochloromethane	Oral and dermal: 0.084	
Tribromomethane	Oral and dermal: 0.0079	
*IUR*—Inhalation unit risk (μg m^−3^)^−1^		[4,13,14]
Trichloromethane	0.000023	
Bromodichloromethane	0.000037	
Dibromochloromethane	0.000024	
Tribromomethane	0.0000011	

^a^ Triangular distribution.

**Table 2:** Summary of water quality analysis.

Parameter	Concentration Range (Min–Max)
pH	6.8–7.5
TOC (mg L^−1^)	0.397–2.053
Free chlorine residual (mg L^−1^)	0–0.6
Fluoride (mg L^−1^)	0.014–0.49
Bromide (mg L^−1^)	0.95–1.35
Chloride (mg L^−1^)	2.48–18.97
Nitrate (mg L^−1^)	0.029–2.50
Trihalomethanes (µg L^−1^)	
TCM	1.7–15.24
BDCM	3.55–23.29
DBCM	4.84–25.52
TBM	1.6–6.57

#### 2.4.1. Ingestion Route

The intake dose through the ingestion of water can be calculated as:

(2) $${CDI}_{ing,i}=\frac{{Cw}_{i}\times IR\times EF\times ED\times CF}{BW\times AT}$$

where, *CDI_ing,i_* is the chronic daily intake of *i*th THM through ingestion (mg kg^−1^ day^−1^), *Cw_i_* is the concentration of *i*th THM in water (μg L^−1^), *IR* is the ingestion rate of drinking water (L day^−1^), *EF* is exposure frequency (days year^−1^), *ED* is exposure duration (year), *CF* is the conversion factor from μg to mg (0.001), *BW* is body weight (kg), and *AT* is averaging time (days).

#### 2.4.2. Dermal Contact Route

Chronic daily intake through dermal absorption of THM can be determined using the following equation:

(3) $${CDI}_{der,i}=\frac{{Cw}_{i}\times SA\times Pd\times t\times F\times EF\times ED}{BW\times AT}$$

where, *CDI_der,i_* is the chronic daily intake of *i*th THM through dermal absorption (mg kg^−1^ day^−1^), *Cw_i_* is the concentration of *i*th THM in water (μg L^−1^), *SA* is the skin surface area (m^2^), *Pd* is the THM permeability of human skin (m min^−1^), *t* is the showering duration (min events^−1^), and *F* is the showering frequency (events day^−1^).

#### 2.4.3. Inhalation Route

Exposure to shower stall/bathroom air that contains THM makes humans susceptible to risk due to inhalation of THM. A recent update to the USEPA guidelines recommends referring to Part F; the supplemental guidelines for inhalation risk assessment [31]. This method recommends calculating the exposure concentration (EC) of THM in the shower air (Equation (4)).

(4) $${EC}_{i}=\frac{{Cair}_{i}\times ET\times EF\times ED}{AT}$$

where *Cair_i_* is the concentration of *i*th THM in shower air (μg m^−3^), *EC_i_* is the exposure concentration of *i*th THM in the air (μg m^−3^), *ET* is the exposure time (h day^−1^), and *AT* is the averaging time (h).

To determine the CR, inhalation unit risk (IUR) data of each THM can be used as shown below.

(5) $${CR}_{i}={EC}_{i}\times {IUR}_{i}$$

where *CR_i_* is the inhalation cancer risk and *IUR_i_* is the inhalation unit risk associated with *i*th THM ((μg m^−3^)^−1^) (Table 1). In inhalation risk models, it is common to assume the boundary condition as initial THM concentration in the shower stall (before the showering event) equal to zero [11,14,15,28]. Therefore, such models are limited to the shower/bathrooms of the first category population where fewer people share the facility, and hence, successive showers are unlikely to occur. At the same time, they fail to represent the common shower stalls used by many people, such as the ones in student hostels/dormitories and public gymnasiums/swimming pools/restrooms, where several successive baths can happen. Hence, a modified method was adopted for inhalation risk assessment as described below.

### 2.5. Modified Inhalation Risk Model

In the case of the second category population, common shower stalls are installed in student hostels where successive showering is frequent, notably in the mornings and evenings. A cumulative increase in bathroom air THM concentration is expected with successive showering events, and hence, in the inhalation risk. To address this, the widely used Little’s model with few changes followed [27]. As per (Little, 1992) [32], the THM concentration (*Cair*) that one can be exposed to for a shower duration (*t*) is:

(6) $${Cair}_{i}=\frac{C_{0,i}+C_{t,i}}{2}$$

where, *Cair_i_* is the estimated concentration of *i*th THM (μg L^−1^), and *C*_0,*i*_ and *C*_0,*i*_ are the before and after concentrations of *i*th THM in the shower stall (μg L^−1^), respectively. *C*_0,*i*_ is calculated as:

(7) $$C_{t,i}=(1-e^{-bt})\times (\frac{a}{b})$$

where,

(8) $$b=\frac{1}{Vs}\{(\frac{Qw}{H})\times (1-e^{-N})+Qg\}$$

(9) $$a=\frac{1}{Vs}\{Qw\times Cw\times (1-e^{-N})\}$$

*Vs* is the volume of the bathroom (L), *Qw* is the water flow rate in the bathroom (L min^−1^), *H* is unitless Henry’s constant at 40 °C for each THM, *Qg* is the airflow rate in the shower (L min^−1^), and *N* is the non-dimensional overall mass transfer coefficient. *N* can be calculated as [14]:

(10) $$N=\frac{KoLA}{Qw}$$

where *KoLA* is the overall mass coefficient of each THM (L min^−1^) (Table 1).

For the first shower, *C_0,i_* can be considered as zero, and hence, *Cair_i_* = ½ *C*_0,*i*_. Assuming back-to-back showering events with no time interval between showers, the second bath will have the *Cair_i_* of the first bath as *C_0,i_* and the subsequent *Cair_i_* = ¾ *C*_0,*i*_. Therefore, for the nth showering event:

(11) $${Cair}_{i}=\frac{2^{n}-1}{2^{n}}\times C_{t,i}$$

Inhalation risk for both population categories was calculated by substituting Equation (9) in Equation (4). For population A, only the first shower was considered for inhalation CR. For population B, up to 10 successive showering events (S1–S10) were considered for inhalation CR.

### 2.6. Effect of Water Temperature on Trihalomethane Concentration

Both dermal and inhalation risk models require adjustment for the effect of water temperature, as it is common practice to mix hot and cold water while bathing. Ingestion risk, however, can be disregarded, since cold or room temperature water is often preferred for drinking. The phase change of THM from water to air is more pronounced at higher temperatures, as these compounds are more volatile, leading to an increase in their concentration in shower air. Additionally, heating water can result in an increase in THM production in the presence of residual chlorine [33]. To address this, Chowdhury and Champagne (2009) [26] developed a model that takes into account THM production during mixing and showering, THM concentration in the cold and mixing water, mixing proportions, and the resulting temperature change. As per this model, THM concentration in the heated water was calculated as

(12) $${Chw}_{i}={Cw}_{i}\times e^{(kh-kc)\times t}$$

where *Chw_i_* is the concentration of *i*th THM in heated water (μg L^−1^), *Cw_i_* is the THM concentration in cold water, *t* is the showering duration (min), and *kh* and *kc* are the rate of THM production for heated and cold water (min^−1^), respectively. *k* for the temperature T (°C) is determined as shown below [26]:

(13) $$k=0.0011e^{0.0407T}$$

Using Equations (10) and (11), the THM concentration in the hot and cold-water mix was calculated. In all calculations of dermal as well as inhalation risks, *Chw* was considered instead of *Cw*.

### 2.7. Adjustments in Population and Location-Based Parameters

Computational models consider population characteristics such as life expectancy, body weight, and body surface area as well as location-specific details such as water temperature, ventilation of shower stalls, and tap water flow rate. These data were acquired from previous studies or government agency reports and were incorporated into the models. As body surface area (*SA*) varies over populations, in the current study, it was calculated for the Indian population [1,30].

(14) $$SA=\frac{4\times BW+7}{BW+90}$$

where *BW* is the body weight (kg).

In order to account for the higher susceptibility to toxic compounds with early-age exposure, age-dependent adjustment factors are commonly incorporated into exposure models. In the present study, the population primarily consisted of university residents who were 18 years of age or older. The USEPA threshold for no age adjustment is 16 years; therefore, no modifications were made for age adjustment in the current study [34]. The CR assessment is based on lifetime exposure, which assumes that the lifetime of the receptor is the exposure duration. Although the student community in this study stays on campus for a relatively short period of 4 to 5 years compared to the category A population, which stays on campus for a longer period, the same exposure duration (*ED*) was selected for both populations to allow for better comparison of CR. The selected lifetime exposure duration is adjusted for any population that is exposed to THM through continuous showering. The minimum, most likely, and maximum limits of shower duration were determined based on observations from the USEPA study. Several shower studies conducted throughout the U.S. reported that the average duration of a single shower event ranged from 6 min to 10.4 min, with an average frequency of five showering events per week [13]. The cold-water temperature range (10 to 20 °C) was chosen based on the range of winter water temperatures at the study location. The hot water temperature range of 35–45 °C was selected based on previous studies [16]. The updated CSF and IUR values for respective THMs were obtained from the IRIS website of the USEPA [4]. In the absence of dermal and inhalation factors, the oral CSF factor was considered for the dermal pathway, while the new IUR values were used for the inhalation pathway [9,11]. Input parameters for all three exposure routes were considered for both females and males (Table 1), and the averages of both genders for each exposure route were expressed as the CR.

## 3. Results and Discussion

### 3.1. Water Quality and Trihalomethane Concentration

The results of the water quality analysis and THM concentrations are given in Table 2. Total THM were in the range of 30.22 ± 14.45 µg L^−1^ with a maximum of 70.65 µg L^−1^, which was within the USEPA maximum contamination limit of 80 µg L^−1^. There was no correlation between the water quality parameters and THM, except for free residual chlorine, which was positively correlated with total THM (*p* < 0.05, R^2^ = 0.63). TCM concentrations followed the three-parameter Weibull distribution and other THM followed a normal distribution.

### 3.2. Risk through Ingestion and Dermal Routes

**Figure 2:**
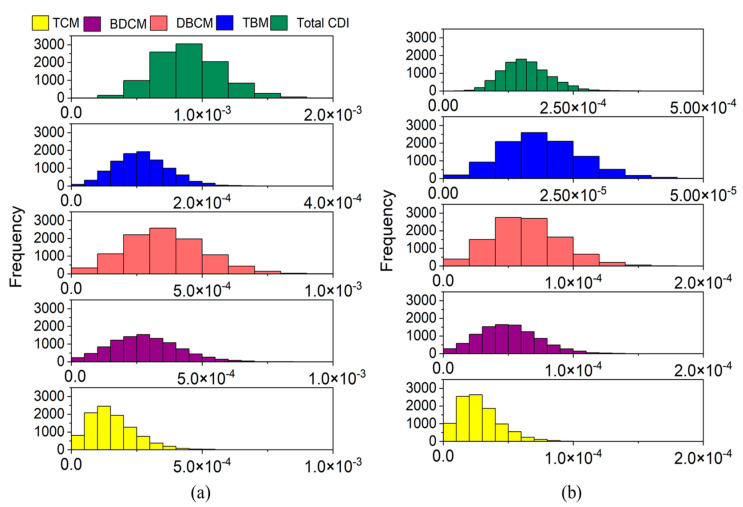
Frequency distribution of chronic daily intake of THM (mg kg^−1^ day^−1^) through (**a**) ingestion and (**b**) dermal contact. TCM: trichloromethane, BDCM: bromodichloromethane, DBCM: dibromochloromethane, TBM: tribromomethane, and CDI: chronic daily intake.

Ingestion resulted in an average CDI of 8.95 × 10^−4^ mg kg^−1^ day^−1^, whereas the dermal pathway was estimated to have an average CDI of 1.59 × 10^−4^ mg kg^−1^ day^−1^. Higher CDI indicates a higher risk for human health. Therefore, in the current study, the ingestion pathway was found to result in higher human health issues than dermal risk. The contributions of individual THM to ingestion as well as dermal pathway are shown in Figure 2a,b. In both cases, DBCM contributed to the total CDI, with an average of 3.55 × 10^−4^ mg kg^−1^ day^−1^ through ingestion and 6.33 × 10^−5^ mg kg^−1^ day^−1^ through dermal contact. This accounted for almost 40% of the total CDI for both routes. Furthermore, DBCM intake was 2.2 times more than that of TCM, 1.3 times more than that of BDCM, and 3.3 times more than that of TBM. For both routes, BDCM was the second highest, with almost 30% of total CDI, followed by TCM (17–18%) and TBM (12%).

**Figure 3:**
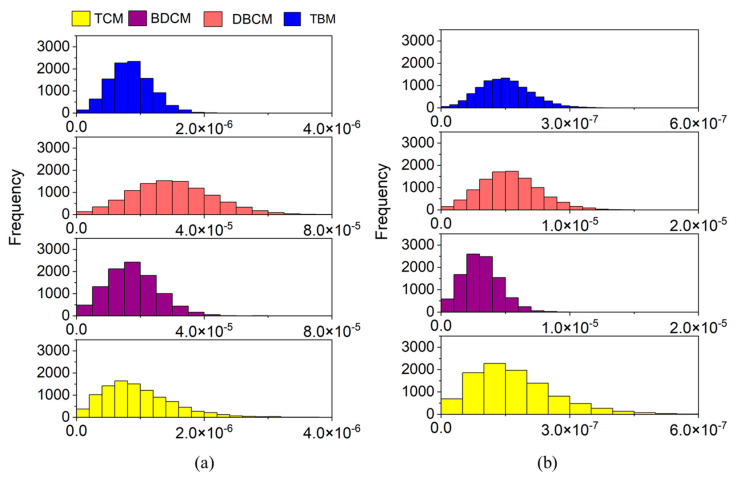
Frequency distribution of cancer risk of THM through (**a**) ingestion and (**b**) dermal contact. TCM: trichloromethane, BDCM: bromodichloromethane, DBCM: dibromochloromethane, and TBM: tribromomethane.

The CR through ingestion and dermal routes were maximum at 1.28 × 10^−4^ and 2.45 × 10^−5^, respectively, with an average of 4.87 × 10^−5^ ± 1.64 × 10^−5^ (Figure 3a) and 8.69 × 10^−6^ ± 2.91 × 10^−6^ (Figure 3b), respectively. Thus, as seen from the CDI results, ingestion was found to impart more CR than dermal contact. DBCM was responsible for around 60% of ingestion as well as dermal CR, followed by BDCM (35%) and TCM or TBM (2% each) (Figure 3a,b). The CR from DBCM was almost 32 times and 35 times higher than that of TCM and TBM, respectively. Meanwhile, BDCM-CR was almost 1.7 times lower than DBCM-CR. CDI and CR patterns observed in the present population were similar to the results of previous studies based on populations from Bangladesh, Canada, and Dhahran [11,15].

### 3.3. Inhalation Route

#### 3.3.1. Inhalation Risk from Private Shower Rooms

**Figure 4:**
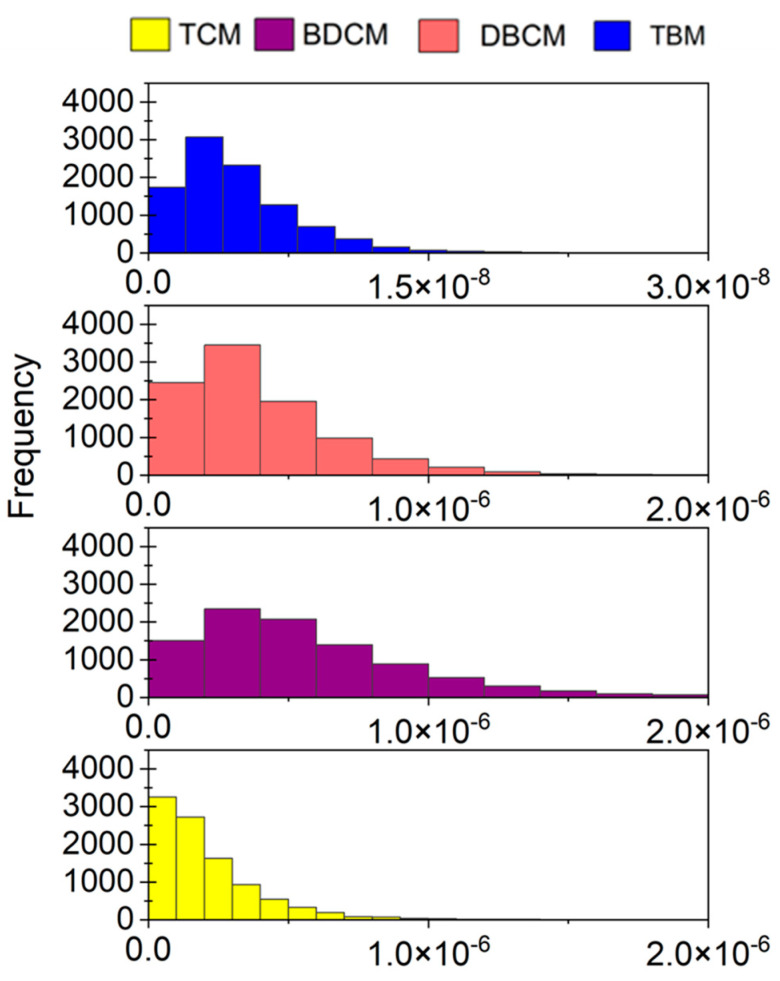
Frequency distribution of cancer risk through inhalation in the case of private shower rooms. TCM: trichloromethane, BDCM: bromodichloromethane, DBCM: dibromochloromethane, and TBM: tribromomethane.

The frequency distribution of inhalation CR associated with each THM in the case of private shower rooms is shown in Figure 4. An average of 1.10 × 10^−6^ ± 6.764 × 10^−7^ and a maximum possible risk of 7.21 × 10^−6^ was determined for inhalation. This was less than the ingestion and dermal contact CRs. The calculated CR indicates a chance of about one in a million cancer cases. Unlike in the case of ingestion and dermal routes, BDCM contributed the most to total inhalation CR (47.4%), followed by DBCM (33.3%), and TCM (18.6%) (Figure 4).

TBM added less than 1% to the total inhalation CR, which can be attributed to the low IUR (Table 1) and its relatively low concentration in water. Furthermore, the CR of BDCM was almost 2.5 times higher than TCM, 1.4 times higher than DBCM, and 114 times higher than TBM. The only study that used the USEPA guidelines Part F for inhalation risk was by Téllez Tovar and Rodríguez Susa, (2020) [14]. In a comparison of old and new methods, a slightly low CR was observed for the new method. Furthermore, the probability distributions of both methods for both genders followed similar patterns.

#### 3.3.2. Inhalation Risk from Common Shower Rooms

**Figure 5:**
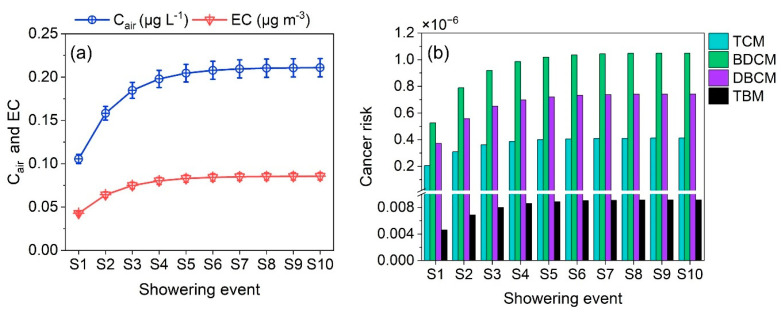
Incremental effect of continuous showering events in common washrooms. (**a**) Increase in total THM concentration in the shower room air (C_air_) and exposure concentration (EC); (**b**) total inhalation risk through continuous showering events. TCM: trichloromethane, BDCM: bromodichloromethane, DBCM: dibromochloromethane, TBM: tribromomethane, and S1–S10: shower event 1 to 10.

The inhalation CR of common shower rooms for up to S10 was assessed using the modified model. The incremental concentration of THM in the shower room air and corresponding ECs were evaluated for calculating the CR (Figure 5). Both *EC* and *C_air_* increased for the first few events and at slower rates for further events, resulting in a logarithmic trend for both parameters. The equation of *C_air_* for the showering event ‘S’ was *C_air_* = 0.0438 ln(S) + 0.1239. For EC, it was *EC* = 0.0178 ln(S) + 0.0502. Using these equations, THM concentration in the shower room air and EC for any showering events can be calculated for similar populations that are exposed to comparable THM concentrations.

The inhalation CR for successive showers followed a similar trend as EC and *C_air_*. The S2 was estimated to pose a 50% increase in inhalation CR than the first shower (Figure 5). Subsequently, a 75% increase by the third (S3), and around a 95% increase by the fifth shower (S5) (Table 3). Thereafter (S6 onwards), the CR slowly increased. By S9, the increase in EC reached 100%, i.e., two times greater than the first shower.

It is important to note that, during the first shower, the CR was almost the USEPA limit for negligible risk (1 × 10^−6^) and it was almost double for S7 onwards. The result indicates that inhalation of S7 alone can result in CR of 2 × 10^−6^. This trend is comparable to the study of Chowdhury et al. (2020) [16], where they observed 1.7%, 26.5%, and 49.9% exceedance probabilities for the USEPA threshold for the second, fifth, and ninth showers. For comparing the impact of individual THM in successive showering events, the minimum (10th percentile), mean, and maximum (90th percentile) of their inhalation CRs are listed in Table 3. The order of individual THM to total inhalation was BDCM > DBCM > TCM > TBM, with TBM as negligibly low CR (Figure 5).

### 3.4. Total Cancer Risk

**Figure 6:**
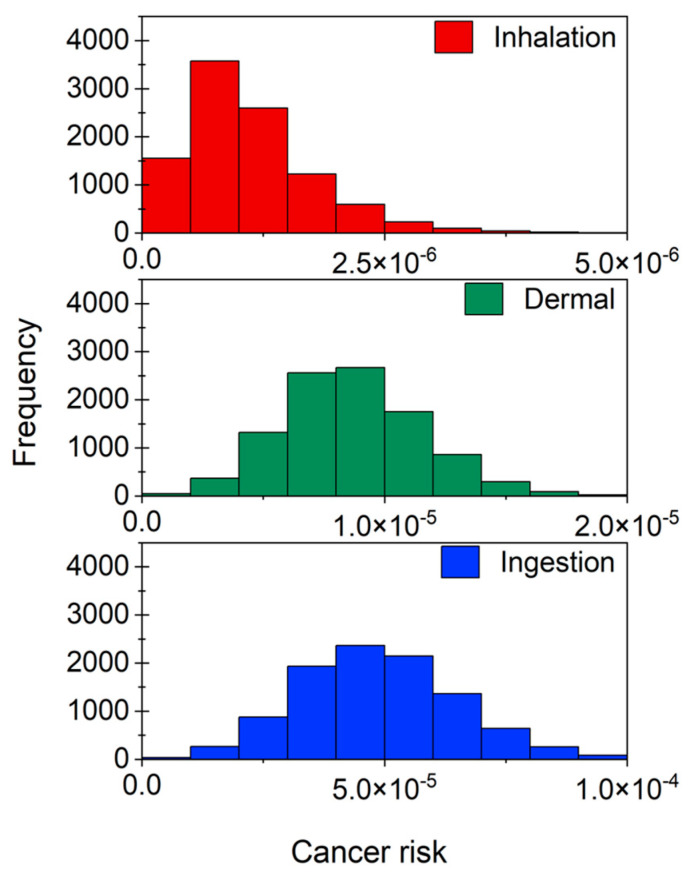
Frequency distribution of total cancer risk through ingestion, dermal contact, and inhalation routes for private shower rooms.

An average CR of 5.85 × 10^−5^ ± 1.67 × 10^−5^ was estimated for private shower rooms, indicating the possibility of almost 58.5 cases per million (Table 3). The 10th and 90th percentiles of CR were 3.81 × 10^−5^ and 8.03 × 10^−5^, respectively. The maximum CR was 1.35 × 10^−4^. When ingestion contributed almost 83% to the total CR, 15% was through dermal contact and only 2% was through inhalation. The frequency distribution of each exposure route to the total CR is given in Figure 6. Ingestion and dermal contact CRs were respectively 43.9 and 5.6 times higher than the CR through inhalation. The average CRs of each THM species were in the order of DBCM (61%) > BDCM (35%) > TCM (2%) = TBM (2%). Further, the total CR of TCM, BDCM, and DBCM was 1.33 × 10^−6^, 2.07 × 10^−5^, and 3.548 × 10^−5^, respectively.

In the case of common shower rooms, the total CR of each showering event was calculated by adding ingestion and dermal contact CR to the respective inhalation CR (Table 3). The total CR of the first shower was 5.85 ×10^−5^, which increased to 5.95 × 10^−6^ by the fifth shower and 5.96 × 10^−6^ by the sixth shower. This implies that the second category population that has common shower rooms poses higher CR compared to the first category population that has access to private shower rooms, even with the same water supply. Out of each THM species, DBCM was estimated with the highest CR, which increased from 3.5 × 10^−5^ for S1 to almost 3.6 × 10^−5^ by S10. The order of the rest of the THM was BDCM > TCM > TBM. Even though the CR of TBM through inhalation was negligible, the total CR associated with TBM was almost equal to the USEPA threshold for the first shower itself.

Table 4 summarizes past studies that have evaluated the CR associated with exposure to THM in household water. Depending on the dominant THM species, different exposure routes have been found to contribute to the CR. In general, ingestion has been found to be the highest contributor to the total CR, while the CR from dermal contact and inhalation routes varies depending on the THM concentration and the population. Clearly, the CR calculated for population A in the current study is in accordance with past studies that had similar THM concentrations.

It is worth noting that the CR due to exposure to THM is affected by population parameters such as exposure duration (life expectancy) and body weight. This difference in CRs is evident from the past studies listed in Table 4. For instance, the THM concentration in tap water in Saudi Arabia and Canada is relatively lower than that of the current study, as well as in Bangladesh, Iran, and many other countries. However, the calculated CR for these two populations is comparatively high despite the negligible concentration of THM. This could be due to the higher life expectancy and body weight of these populations, which are a result of their developed and improved living situations.

### 3.5. Effect of Ventilation and Shower Duration on Cancer Risk

**Figure 7:**
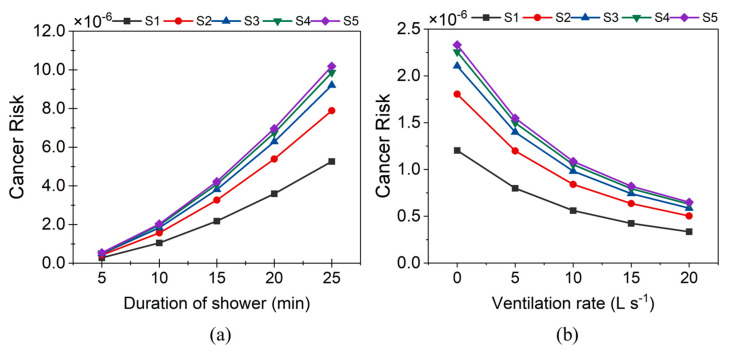
Effect of (**a**) showering duration and (**b**) ventilation rate on total cancer risk.

Two parameters in the inhalation CR assessment that were subjected to great variability are shower duration and ventilation rate in the shower rooms. Shower duration is a habit that varies from one individual to another. While there are standard minimum ventilation requirements for shower rooms, many shower stalls in the study area lacked exhaust fans or ventilation windows. Therefore, to check the effect of ventilation and shower duration on inhalation CR, Equation (7) was simulated with different levels for variables *t* and *Q_g_* with the rest of the parameters unchanged. CR of up to S5 was assessed with shower durations varying from 5 min to 25 min and ventilation rates varying from 0 L s^−1^ (closed shower room with no ventilation) to 20 L s^−1^ (Figure 7).

With increasing shower duration, the CR was found to exponentially increase (*p* = 0.001, R^2^ = 0.98). For S1, S3, and S5, the CR for various shower durations was estimated as follows.

(15) $${CR}_{1}=2\times 10^{-7}\times e^{0.142t}$$

(16) $${CR}_{3}=3\times 10^{-7}\times e^{0.142t}$$

(17) $${CR}_{5}=4\times 10^{-7}\times e^{0.142t}$$

where, *CR*_1_, *CR*_3_, and *CR*_5_ are the cancer risk associated with the first, third, and fifth showering events, and *t* is the showering duration (min).

The S2 of any duration was determined to pose 1.5 times higher inhalation CR than the respective S1. Similarly, the CR of S3, S4, and S5 was 1.7, 1.8, and 1.9 times higher than the CR of S1, respectively. The CR for S1 increased from 2.8 × 10^−7^ to 5.26 × 10^−5^ (18.8 times) for an increase in shower duration from 5 min to 25 min. Furthermore, the CR associated with 5 min was less than the USEPA threshold (Figure 7a), even by S5. Whereas for 10 min, the CR of S1 itself was 1 × 10^−6^. In the case of a 25-min shower, the CR estimated for S1 was 5.25 × 10^−6^. This was equal to the CR associated with the 20-min long S2 and was a little higher than the CR associated with the 15-min long S3. The inhalation CR of S5 for 25-min long successive showers was as high as 1.02 × 10^−5^. The total CR, in this case, was as high as 6.742 × 10^−5^ with ingestion and dermal contact CRs added. Previous studies have reported similar trends [16].

Increasing the ventilation inside the shower room substantially reduced the CR (*p* = 0.010, R^2^ = 0.91) (Figure 7b). When the ventilation rate increased from 0 to 5 L s^−1^, the CR of the corresponding S1 reduced from 1.2 × 10^−5^ to 7.9 × 10^−7^. For 20 L s^−1^, this CR was further reduced to 3.35 × 10^−7^. Equations (16) and (17) show the effect of the ventilation rate (*Qg*) in the range of 0–20 L s^−1^ on inhalation CR for the successive showering events.

(18) $${CR}_{1}=1\times 10^{-6}\times e^{-0.064Qg}$$

(19) $${CR}_{3,5}=2\times 10^{-6}\times e^{-0.064Qg}$$

At no ventilation, the CR of S1 to S5 was above 1 × 10^−6^. However, the CR of S1 at 5 L s^−1^ and that of S1, S2, and S3 at 10 L s^−1^ were less than 1 × 10^−6^. The CR of S1 to S5 was negligible for a ventilation rate of 15 L s^−1^ and above. These results indicate that a well-ventilated shower stall can significantly reduce the inhalation CR. This observation is significant in places where inhalation is the major route for THM risk, such as swimming pools, chlorinated disinfectant manufacturing plants, cleaning indoors using bleach, and disinfection workers who spray chlorinated products [39,40,41].

Previous studies have reported no THM in the supplied tap water but in indoor air [3,42]. The high volatility rate of THM, especially that of TCM can be attributed to this. Results of the current study emphasize the significance of proper ventilation in showering facilities to mitigate the risk of inhalation exposure. This can be achieved through the incorporation of windows and ventilators to enable natural air exchange or through the installation of exhaust fans in high-risk areas. The inhalation CR can be further reduced by allowing sufficient time between successive showering events and ensuring proper ventilation. The model used in this study accounted for back-to-back showering events, which is common during rush hours, thus underscoring the need for adequate ventilation in such scenarios. The findings of this study can guide water treatment facilities to reduce THM concentrations and mitigate health risks associated with exposure to THMs.

## 4. Conclusions

Ingestion and dermal contact intakes of the populations were estimated to be 8.95 × 10^−4^ mg kg^−1^ day^−1^ and 1.59 × 10^−4^ mg kg^−1^ day^−1^ (mean), respectively. This resulted in a CR of 4.873 × 10^−5^ through ingestion and 8.69 × 10^−6^ through dermal contact. Population A, which had access to private shower rooms, was exposed to a total CR of 5.83 × 10^−5^, which included the inhalation CR of 1.1 × 10^−6^. DBCM posed the most risk (60%) to both ingestion and dermal contact, while BDCM (47.4%) contributed the highest inhalation risk. TBM had a negligible impact on inhalation risk, mainly because of the relatively low concentration in the supplied water and because of the low IUR value.

Continuous showering in common stalls increased the risk through inhalation with each successive event. The inhalation CR of the third shower was 75% greater than the first shower, and the fifth had a 95% increase than the first shower, and by the nineth showering event, the inhalation CR was double that of the first event. Subsequently, the total CR also increased; it was 5.95 × 10^−5^ for the fifth event. While the results of this study did not show a significant increase in CR for successive shower events compared to single events, it is important to note that CR is directly proportional to the concentration of THM in the water. Therefore, the CR may vary for different types of water. However, the methodology developed in this study could be used to investigate the impact of successive shower events on inhalation CR in other contexts. Each exposure route was responsible for a CR that was higher than the USEPA threshold for negligible risk, indicating the THM levels in the water need to be controlled. However, an increase in ventilation rate significantly reduced the THM concentration in the shower room air, and thereby, subsequent inhalation risk. The introduction of 5 L s^−1^ in a closed shower room reduced the inhalation CR by 1.5 times and a ventilation rate of 20 L/s further reduced the inhalation CR by almost 3.5 times. Higher shower duration led to high exposure to inhalation risk. The first showers of 5 min and 25 min had CRs equal to 0.27 × 10^−6^ and 5.25 × 10^−6^; all five successive showers with 5-min durations had CRs less than the USEPA threshold, whereas the first shower of 10 min crossed this limit. The results of the present study underscore the significance of adequate ventilation in shower facilities and provides valuable insights into the water treatment industry for devising strategies to mitigate THM formation and subsequent inhalation risk for consumers.

## Figures and Tables

**Table 3:** 10th, 50th, and 90th percentile values and total cancer risk per THM compound for successive showering events in common showering rooms.

THM Species	Cancer Risk (Minimum, Mean, Maximum) × 10^−6^							Total Cancer Risk (Mean) × 10^−6^				
	Inhalation					Ingestion	Dermal Contact	S1 ^a^	S3	S5	S7	S10
	S1 ^b^	S3	S5	S7	S10							
TCM	0.03	0.05	0.06	0.06	0.06	0.34	0.06	1.33	1.49	1.53	1.54	1.54
	0.21	0.36	0.40	0.41	0.41	0.96	0.17					
	0.45	0.79	0.87	0.89	0.90	1.69	0.30					
BDCM	0.08	0.14	0.15	0.16	0.16	6.83	1.23	20.71	21.11	21.20	21.23	21.24
	0.53	0.92	1.02	1.04	1.05	17.13	3.05					
	1.07	1.88	2.08	2.13	2.14	27.83	4.96					
DBCM	0.08	0.14	0.15	0.16	0.16	13.69	2.45	35.49	35.77	35.84	35.85	35.86
	0.37	0.65	0.72	0.74	0.74	29.80	5.32					
	0.74	1.30	1.43	1.47	1.48	46.59	8.30					
TBM	0.001	0.002	0.002	0.002	0.002	0.431	0.075	0.999	1.003	1.003	1.004	1.004
	0.005	0.008	0.009	0.009	0.009	0.846	0.149					
	0.009	0.015	0.017	0.018	0.018	1.283	0.228					
Total	1.11	1.94	2.15	2.20	2.21	48.73	8.69	58.53	59.36	59.57	59.62	59.64

^a^ Results of S1 also represents the total of private shower rooms. ^b^ S1–S10 showering event 1–10.

**Table 4:** THM concentration in various water supply systems and their associated cancer risk.

Reference	Location	Trihalomethane Concentration (µg L^−1^) ^a^				Cancer Risk		
		TCM	BDCM	DBCM	TBM	Ingestion	Dermal	Inhalation
[35]	Jharkhand and West Bengal, India	3.92–532.64	0.0–315.2	0.0–187.07	9.78–1854	3.93 × 10^−5^–3.19 × 10^−3^	<10^−8^	6.02 × 10^−5^–3.36 × 10^−3^
[36]	Eastern India	223–461	2–13	2–13	231–484	2.37 × 10^−4^	5.76 × 10^−9^	negligible
[37]	Karachi, Pakistan	8.4–167.3	0.19–3.3	0.22–1.9	0	1.10 × 10^−5^	4.42 × 10^−5^	6.68 × 10^−5^
[15]	Dhaka, Bangladesh	Total THM = 20.2–439.2 including TCM = 6.25–134.06.				4.08 × 10^−5^	5.45 × 10^−12^	9.57 × 10^−10^
[11]	Toronto, Canada	1.4–10.7	1.0–5.8	0.8–3.6	0–0.5	9.0 × 10^−6^	3.0 × 10^−6^	3.0 × 10^−6^
	Dhahran, Saudi Arabia	1.2–6.1	0.5–3.4	0.0–1.2	0–0.7	3.0 × 10^−6^	1.0 × 10^−6^	1.0 × 10^−6^
[9]	Jiangsu Province, China	4.8–20.4	1.7–12.7	4.2–12.0	0.2–7.2	5.81 × 10^−6^–1.67 × 10^−5^	2.82 × 10^−6^–7.98 × 10^−6^	2.24 × 10^−6^–1.54 × 10^−5^
[18]	Tabriz, Iran	0.0–101	0.0–601	0.0–118	0.0–79	2 × 10^−4^	2.35 × 10^−5^	3.43 × 10^−5^
[14]	Bogotá, Colombia	31.25	0.0	4.01	4.09	_ ^b^	_	18 × 10^−6^–48 × 10^−6^
[38]	All over Egypt	Total THM = 29.07–86.01				Total Cancer risk = 1.0 × 10^−6^–42.2 × 10^−6^		
Current study ^c^	West Bengal, India	1.7–15.24	3.55–23.2	4.84–25.5	1.6–6.57	48.73 × 10^−6^	8.69 × 10^−6^	1.11 × 10^−6^

^a^ ‘0’ indicate below detection limit; ^b^ only inhalation risk from showering was calculated; ^c^ to compare with the above studies, only cancer risk of population A is shown.

## Data Availability

The data presented in this study are available on request from the corresponding author.
